# Reordering Hierarchical Tree Based on Bilateral Symmetric Distance

**DOI:** 10.1371/journal.pone.0022546

**Published:** 2011-08-04

**Authors:** Minho Chae, James J. Chen

**Affiliations:** 1 Division of Personalized Nutrition and Medicine, National Center for Toxicological Research, U.S. Food and Drug Administration, Jefferson, Arkansas, United States of America; 2 Cecil H. and Ida Green Center for Reproductive Biology Sciences, University of Texas Southwestern Medical Center at Dallas, Dallas, Texas, United States of America; 3 Graduate Institute of Biostatistics & Biostatistics Center, China Medical University, Taichung, Taiwan; King's College London, United Kingdom

## Abstract

**Background:**

In microarray data analysis, hierarchical clustering (HC) is often used to group samples or genes according to their gene expression profiles to study their associations. In a typical HC, nested clustering structures can be quickly identified in a tree. The relationship between objects is lost, however, because clusters rather than individual objects are compared. This results in a tree that is hard to interpret.

**Methodology/Principal Findings:**

This study proposes an ordering method, HC-SYM, which minimizes bilateral symmetric distance of two adjacent clusters in a tree so that similar objects in the clusters are located in the cluster boundaries. The performance of HC-SYM was evaluated by both supervised and unsupervised approaches and compared favourably with other ordering methods.

**Conclusions/Significance:**

The intuitive relationship between objects and flexibility of the HC-SYM method can be very helpful in the exploratory analysis of not only microarray data but also similar high-dimensional data.

## Introduction

Recent developments of high-throughput technology such as genotyping arrays have generated a wealth of high-dimensional data. In order to analyze this huge amount of data as a whole set, various clustering methods [1]–[3] are used where a set of objects are grouped into clusters in an unsupervised approach *i.e.*, no *a priori* knowledge is assumed. Objects within the same cluster should be similar to one another and dissimilar to the objects in other clusters. Through clustering, hidden data patterns can be revealed, *e.g.*, samples of genes expressed under varying conditions can be assigned to biologically meaningful groups based on their gene expression levels over a set of experiments.

**Figure 1:**
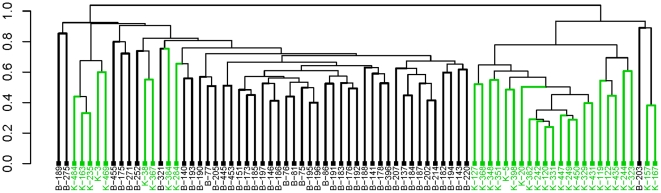
Hierarchical clustering of two tissue types. Malignant breast and kidney samples, n = 45 and n = 32, respectively, are taken from Liu *et al.* data [11] for hierarchical clustering using the average-linkage method with the Pearson correlation distance.

Hierarchical clustering (HC) is one of the most popular clustering methods. Typical hierarchical clustering is agglomerative; beginning with as many clusters as objects, similar clusters are sequentially merged based on some form of distance metrics, *e.g.*, Euclidian distance, until only one cluster remains. For *n* objects, *n*-1 merging steps are needed to produce a tree diagram, also known as a dendrogram. The final dendrogram, however, is not unique because the tree at each merging step can be flipped in either direction. In fact, there exist 2*^n^* ^-1^ possible dendrograms with the same clustering structure. Figure 1 is a dendrogram by HC for microarray data with two different tissue types. While the majority of samples are grouped according to their tissue types, not only are misclustered samples (green branches in the black group and black ones in the green group) found but they are far apart from their proper group. Better grouping can be obtained by simply flipping some internal nodes. This presents a major challenge to users since it is impossible to test all possible forms, especially when the labels, *i.e.*, leaf colors, are unknown. Another drawback of HC stems from the greedy approach of the method. All clustering steps are entirely based on local decisions without any re-evaluation of the clustering. Any initial misbehaving grouping is carried on to the end [4]. Furthermore, the relationship between the elements is lost during the clustering process since clusters rather than individual elements are compared [5]. The resulting tree can be difficult to interpret.

One way to get around the shortcomings of HC is to order the leaves with some heuristics or dimension reduction methods. Gruvaeus and Wainer [6] proposed a method joining two clusters in each merging step such that the endpoints of the clusters are most similar. In Eisen's heuristic of popular heat map [7], average expression level was used in determining the order of leaves. Bar-Joseph *et al.* [8] proposed a fast optimal leaf ordering method that finds a linear ordering of leaves in which the sum of similarities of neighbouring leaves is maximized. Dimension reduction methods can be also used for ordering as in Tien *et al.* [9] where the rank-two ellipse (R2E) [10] was used as en external reference to guide in flipping internal nodes of a tree.

In this paper, we propose a method, HC-SYM, to order the leaves of a binary tree based on their bilateral symmetric distances. The purpose is to put more similar objects in the middle and objects that are more dissimilar in the outside of tree in order to represent the tree in more smooth patterns while keeping the local grouping structure of HC intact. For complex data sets with many objects and classes, this method can be selectively applied according to levels of the tree for flexible exploratory analysis. The approach is illustrated with a public microarray gene expression dataset with varying number of classes. The results are compared to other ordering methods.

## Methods

### Distance measurements between clusters

**Figure 2:**
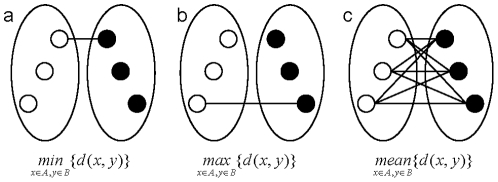
Distance measurements between clusters. Three most widely used inter-cluster distances are (a) single linkage, (b) complete linkage, and (c) average linkage method.

**Figure 3:**
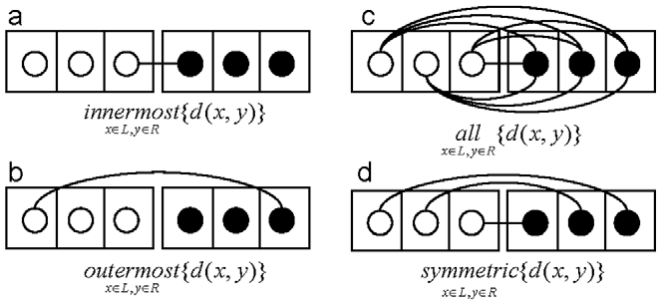
Distance measurements between clusters with linear ordering of objects. Four inter-cluster distance measurements for the left (*L*) and right (*R*) cluster can be considered when taking the order of objects into account; (a) *innermost* linkage, (b) *outermost* linkage (c) *all* linkage, and (d) *symmetric* linkage.

In hierarchical clustering methods, the distance between two clusters can be measured in several ways as a function of the distance between objects in the two clusters. If *x* 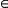 *A*, *y* 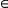 *B*, and *d*(*x*, *y*) = distance metric between objects *x* and *y*, then the distance between *A* and *B* can be measured using one of the following methods: the nearest neighbour or single linkage (Figure 2(a)); the furthest neighbour or complete linkage (Figure 2(b)); or the average distance between all pairs (Figure 2(c)). With these distance measurements, the ordering of objects within each cluster, typically drawn as an oval with objects in them, does not affect the inter-cluster distances. The ordering of objects within a cluster, however, is important since the objects are linearly located in a dendrogram, the visual representation of HC, and it might be more critical for interpretation in a certain study. To order objects using inter-cluster distance, *innermost* , *outermost*, *all*, or *symmetric* pairs are used as shown in Figure 3 where the left and right subtree of a dendrogram are designated as the left (*L*) and right clusters (*R*), respectively: they are not *A* and *B* since changing their locations would result in different inter-cluster distance. This strategy is similar to Figure 2 in that each pairing method has a corresponding linkage: *innermost* to single linkage, *outermost* to complete linkage, and *all* to average linkage. However, *symmetric* pairing has no counterpart linkage.

### Problem defined

Let *L* = {*x*_1_,…, *x_m_*} and *R* = {*y*_1_,…,*y_n_*} (or *L* = {*x_−m_*,…, *x*_−1_} and *R* = {*y*_−n_,…,*y*_−1_} when allowing negative indexing) be left and right clusters whose objects need to be ordered. Our goal is to locate similar *x* and *y* closer in the linear order by using distances between members and their adjacent cluster, *i.e.*, *d*(*x*, *R*) or *d*(*L*, *y*) where *d* is a distance metric. If we use the *all*-linkage approach, we can use the average distances for all members in *L* and *R* and order the members according to their average. In this approach, the time complexity to look up the all *m*+*n* members in *d* is *O*((*m+n*)^2^). Simpler and less computing intensive approach taking linear time to look up is to use only symmetric pairs of the two clusters so that
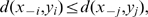
(1)where *i *<* j* for all *i*, *j* 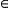
{1..*k*} if *k* is size of the smaller cluster: the distance of inner symmetric pairs are smaller than or equal to the one of outer symmetric pairs. If we define *B*, *bilateral symmetric distance* between *L* and *R*, to be
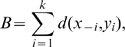
(2)where *k* is the size of smaller cluster as before, then *B* is minimized when condition (1) is met.

If the size of *L* and *R* is equal, the order of all members can be determined purely based on the bilateral symmetric distance. For the unequal size of clusters, some members inevitably do not have their symmetric counterpart. However, they still should be ordered for smooth transition. Ordering can be done based on the distances between the unpaired members to their adjacent clusters, *i.e.*, *d*(*x*, *R*) or *d*(*L*, *y*). Alternatively, the unpaired members can be compared to their own clusters, *i.e.*, *d*(*x*, *L′*) or *d*(*R′*, *y*) when *L′* and *R′* is the set of members determined by the bilateral symmetric distance for *L* and *R*, respectively.

### Algorithms

**Figure 4:**
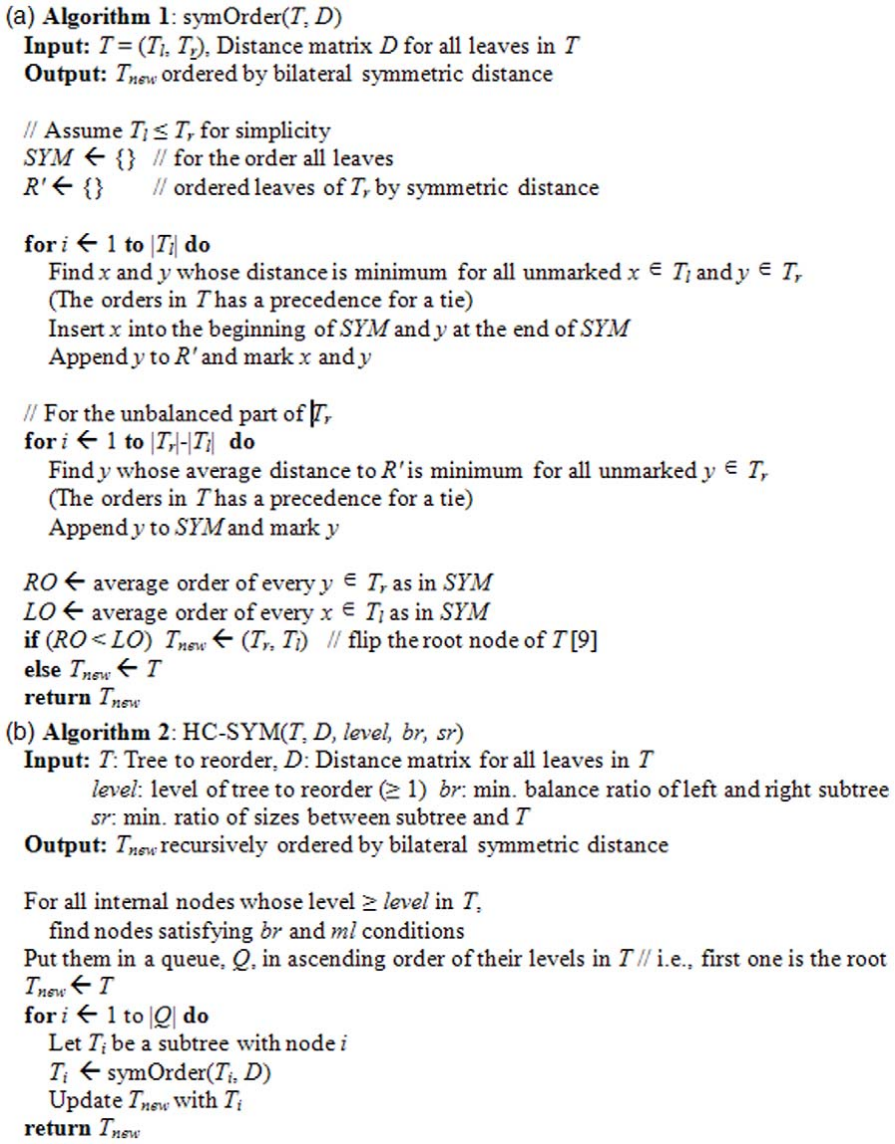
Algorithms. Pseudocodes of the two main functions for the implementation of ordering algorithms based on the *bilateral symmetric distance*; (a) symOrder (b) HC-SYM.

As in Figure 4, two main functions, *symOrder* and *HC-SYM*, are used for implementation of the ordering algorithms based on the bilateral symmetric distance. The former orders the leaves of tree, *T*, given a distance matrix, *D*, according to the symmetric distances of the leaves in the two subtrees of *T*, the left (*T_l_*) and the right (*T_r_*), respectively. First, we order tree only for the balanced part *i.e.*, the number of objects is twice the *min*(|*T_l_*|, |*T_r_*|) when |*T*| denotes the number of leaves in *T*. We fill an order vector, *SYM*, by sequentially selecting the minimum distance of pairs of *x* and *y* where *x* 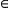 *T_l_* and *y* 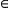 *T_r_*. The result is an order of objects all based on the symmetric distances for the perfectly balanced tree. However, most trees are unbalanced. To order unbalanced part of trees, we use the average-linkage approach where unpaired objects are ordered by the average distances with the already ordered objects in the same cluster. The resulting vector is concatenated with *SYM*. The leaves of *T* are ordered based on *SYM* in which each internal node is flipped only if the average order of the leaves in its right subtree in *SYM* precedes the one in its left subtree (Discussed more detail in Tien *et al.* [9]). The original internal structure of *T* is absolutely preserved because flipping internal nodes do not change the hierarchy of clusters.

*HC-SYM*, on the other hand, is a recursive version of *symOrder* where ordering can be applied to different levels of the tree from top to bottom. Level 1 means the entire tree is ordered using the left and the right subtrees of the root as *T_l_* and *T_r_*, respectively. Level 2 means ordering is done once again using the grand children of the root as *T_l_* and *T_r_* (There are two sets of *T_l_* and *T_r_* here since the root of a binary tree has four grand children nodes). In addition to the *level*, there are two more parameters in *HC-SYM*: (1) *br*, the ratio between *T_l_* and *T_r_*, and (2) *sr*, the minimum ratio of sizes of subtree, *T_l_* or *T_r_*, to *T*. Target nodes are selected according to *level*, *br* and *sr* and they are sequentially run by *symOrder* in a top-down approach; parental nodes are run before their descendant nodes.

## Results and Discussion

### Datasets

We used a public gene expression dataset published by Liu *et al.* [11] for illustration of the proposed ordering method (http://www.samsi.info/200304/dmml/web-internal/bio/data/data_rsvd.xls). The data were human tissue gene expression profile on oligonucleotide microarrays from Affymetrix consisting of 224 genes analyzed in 255 malignant and 249 normal samples as shown in Table 1. A total of 504 samples of eight tissue types including breast, colon, kidney, liver, lung, ovary, prostate, and testis were analyzed. The goal of the data analysis was to group samples according to their biological functions, *i.e.*, tissue types. Two subsets of the data have been analyzed, set A consisting of 77 total malignant breast and kidney tissues, and set B consisting of all 504 samples. Additionally, the yeast cell-cycle data of Cho *et al.* [12] has been included in the analysis [Figure S5].

**Table 1:** Test datasets.

	Tissue type							
Pathology	breast	colon	kidney	liver	lung	ovary	prostate	testis
normal	37	67	37	19	49	5	29	6
malignant	45*	66	32*	20	52	5	29	6
total	82	133	69	39	101	10	58	12

Dataset A:

*(n = 77) and Dataset B: entire 504 samples.

### Performance evaluation

Since cluster membership is determined already by HC, the object of the performance evaluation of our study is not to evaluate quality of typical clustering but rather to evaluate the performance of seriation [13], the suitable linear ordering of objects, without changing the internal structure of a tree. There can be two approaches for this purpose, supervised or unsupervised. Clustering is intrinsically unsupervised learning. However, if we use dataset with the known cluster labels for a testing purpose, we can take a supervised approach in which quality of grouping is measured directly from the linear order of leaves in a tree. For a tree of size *n*, we define *ld*(*x*_1_, *x*_2_) to be a positive *linear distance* for the two leaves *x*_1_ and *x*_2_ belonging to the same class when the leaves are numbered 1 to *n* starting from the left of a tree. So *ld* is 1 when *x*_1_ and *x*_2_ are adjacent and *n*-1 when they are apart the furthest. In order to measure grouping of each class, we define the *seriation score*, *S*(*c*) for a class *c* to be:
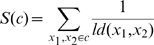
(3)
*S*(*c*) is maximized when all members of *c* are adjacent each other. We also define the *seriation rate*, *SR*, to be the sum of seriation scores for all classes divided by the sum of maximum seriation scores which occurs when all objects are grouped with no error, so that *SR* ranges 0 to 1. Thus, *SR* can be simplified as
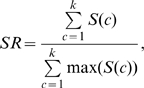
(4)where *k* is the number of total classes.

For the unsupervised approach, however, the scoring measure, *SR*, is not applicable since the class labels are unknown. One possible approach is to use a symmetric proximity matrix. A *n*×*n* symmetric similarity matirx *R* = [*r_ij_*] is called a perfect *Robinson matrix* [14], [15] when all the values in *R* is increasing when moving toward the main diagonal such that *r_ij_* ≤ *r_ik_* if *j* < *k* < *i* (lower triangle) and *r_ij_* ≥ *r_ik_* if *i* < *j* < *k* (upper triangle). Chen [10] proposed generalized anti-Robinson (GAR) scores which is a loss function counting the number of violations of Robinson matrix form for a *n* × *n* permuted proximity matrix *P* = [*p_ij_*] for varying window sizes, *w*, resulting in
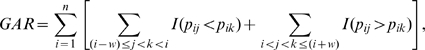
(5)where *I*(.) is an indicator function returning 1 only for violations of Robinson form.

### Comparison to other methods

In addition to the basic hierarchical clustering (HC), we compared HC-SYM to two additional seriation methods; HC-PCA and HC-OLO (optimal leaf ordering). For HC-PCA, the first component of PCA was obtained and was used as an external reference in flipping internal nodes of tree. HC-OLO was available in *seriation* package [13] in R [16]. For visual representation of the analysis, a dendrogram with colored branches was used for the supervised approach. For the unsupervised approach, on the other hand, a color image of the symmetric similarity matrix was used to show *two-way* (both rows and columns) and *one-mode* (sample) data . The default values for HC-SYM was used thoughout the whole analysis (*br* = 0.3 and sr = 0.03).

### Results

**Figure 5:**
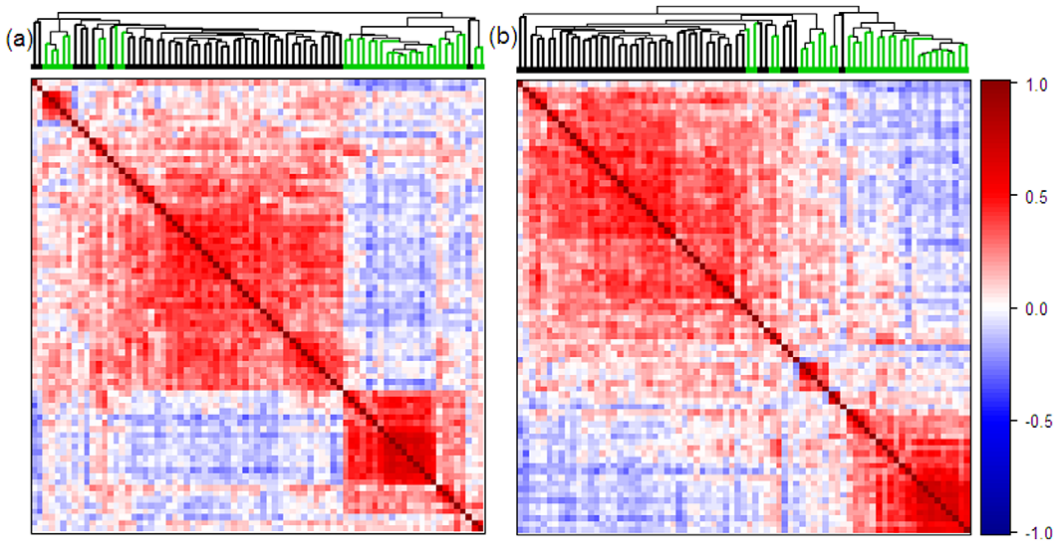
Dendrograms with proximity view for dataset A. Comparison of (a) HC and (b) HC-SYM for dataset A using dendrograms and proximity matrices. HC was performed by the average-linkage method with the Pearson correlation distances. Proximity matrices were drawn with the Pearson correlation coefficients.

**Figure 6:**
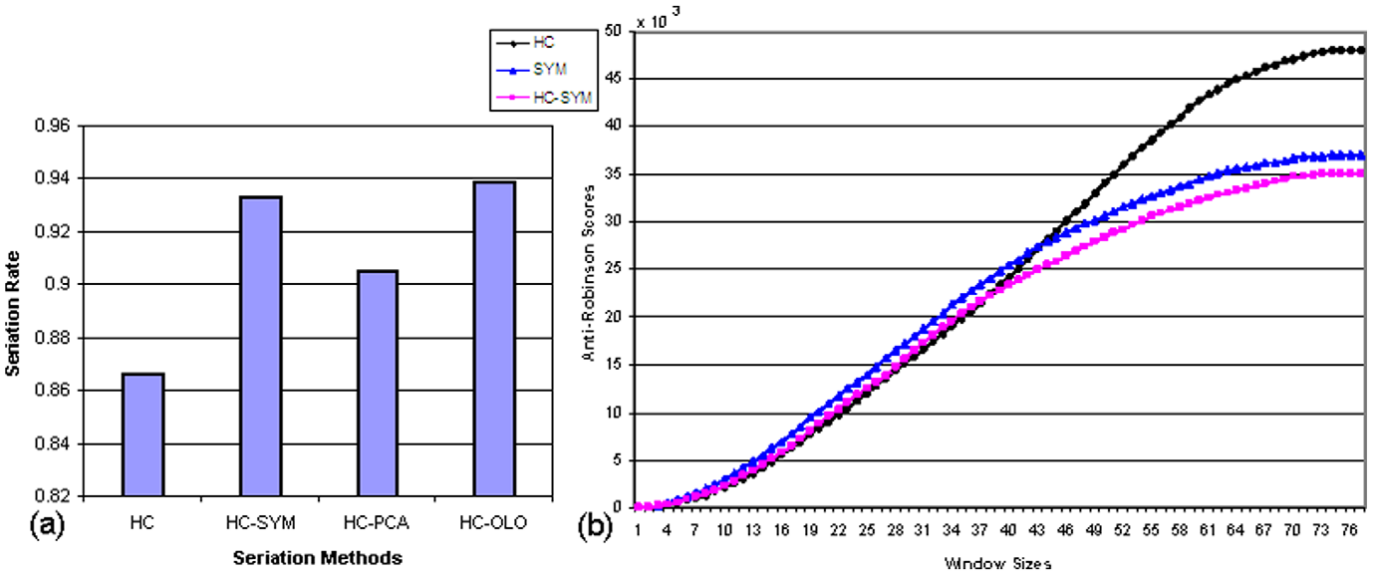
Result for dataset A. Performance measured by (a) Seriation rate (b) Anti-Robinson scores for dataset A.

The result of HC and HC-SYM for dataset A was visually compared in Figure 5. After ordering, improved clustered patterns were clearly noticed in the dendrogram of HC-SYM (5a versus 5b). In addition, the overall shape of the dendroram becomes triangular; loose clusters (long branches) in the middle and tight clusters (short braches) outside. This tendency was also observed in the proximity view of the similarity matrix where the distinction between two clusters were rather fuzzy in the middle and more reddish spots were found when moving towards corners along the diagonal. In the *SR* metrics HC-SYM and HC-OLO showed relatively good performance as shown in Figure 6(a). Figure 6(b) illustrated good local (*i.e.*, window size <37) characteristics of HC and inferior performance on global (*i.e.*, window size >46) perspective. HC-SYM, however, showed relatively strong performance in both smaller and bigger window sizes. In addition, HC-SYM is compared with other methods using anti-Robinson scores. The results are in Figure S1.

**Figure 7:**
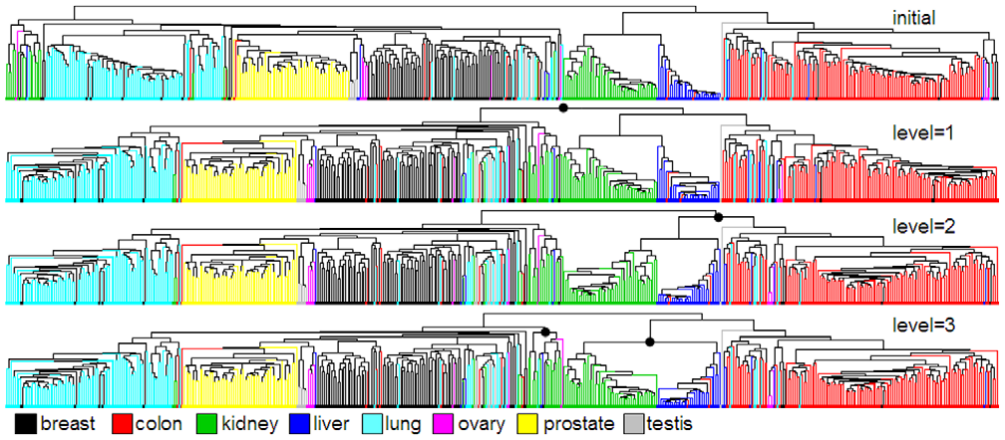
Dendrograms of HC-SYM for dataset B at various levels. HC-SYM was applied at different levels.

**Figure 8:**
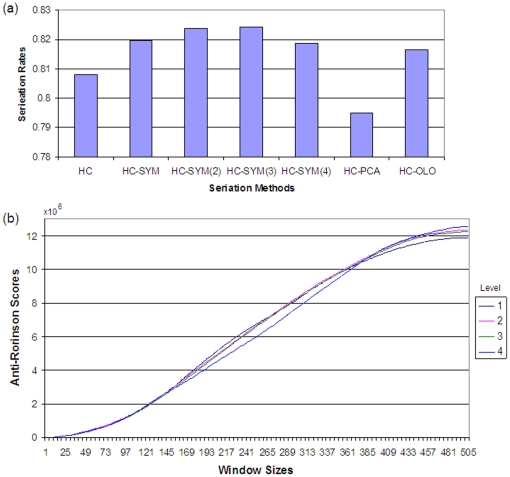
Result for dataset B. Performance measured by (a) Seriation rate (b) Anti-Robinson scores.

Figure 7 illustrates changes in order for dataset B when HC-SYM is applied at different levels where black dots represent the nodes whose left and right subtrees were ordered based on the symmetric distance. Also a triangular shaped dendrogram was observed in most of the selected nodes for ordering. For this complex dataset, HC-SYM showed the best *SR* even at the first level and maximal *SR* was observed at the level four (Figure 8(a)). Figure 8(b) shows the anti-Robinson score decreasing in the mid window sizes and increasing in the bigger window sizes as the level increases; improved local behaviour while sacrificing global behaviour. When compared with other methods by anti-Robinson measure, HC-SYM showed better performance in the global scale and similar local performance with other methods [Figure S2]. As in dataset A, in the proximity view, cells with higher similarity scores were observed more in the corners of clusters [Figure S4] when compared to the result of HC [Figure S3].

### Conclusions

One major drawback of HC is its lack of a systematic ordering method to show global patterns of data. HC-SYM is proposed as one possible remedy where a tree is ordered to minimize the symmetric distances between adjacent clusters. After the ordering, the relationship between objects becomes clearer and the performance of clustering was favourably evaluated with other existing methods. Additionally, HC-SYM approach is flexible as it can be applied at various levels of a tree. This ordering method, especially along with a proper visual aid, proximity view, can be very helpful in the exploratory analysis of not only microarray data but also similar high-dimensional data.

## Supporting Information

Figure S1 **Comparison of Anti-Robinson scores for dataset A.** Anti-Robnison scores by four seriation methods were compared for dataset A.
(TIF)

Figure S2 **Comparison of Anti-Robinson scores for dataset B.** Anti-Robnison scores by four seriation methods were compared for dataset B. The *level* of HC-SYM was 1.
(TIF)

Figure S3 **Dendrogram with proximity view for dataset B using HC.** The result of HC by both dendrogram and color image of similarity matrix.
(TIF)

Figure S4 **Dendrogram with proximity view for dataset B using HC-SYM.** The result of HC-SYM by both dendrogram and color image of similarity matrix. The *level* of HC-SYM was 1.
(TIF)

Figure S5 **Seriation of yeast cell cycle data.** The yeast cell-cycle data of Cho *et al.* [16] containing time-course expression profiles more than 6000 genes at 17 time points was analyzed with the proposed method using 145 genes whose phases have been assigned with a removal of one abnormal time point as suggested by Tamayo *et. al* [4]. (a) Comparison of seriation rates (b) Dendrogram of HC and HC-SYM at level 2. The parameter values were *br* = 0.3 and *sr* = 0.03 for HC-SYM after HC was carried by average-linkage with Pearson correlation distance.
(TIF)
