# Supplementary figures and images for: Reordering Hierarchical Tree Based on Bilateral Symmetric Distance
Source: PLoS One. 2011 Aug 4;6(8):e22546. doi: 10.1371/journal.pone.0022546 (PMC3150382; doi:10.1371/journal.pone.0022546)

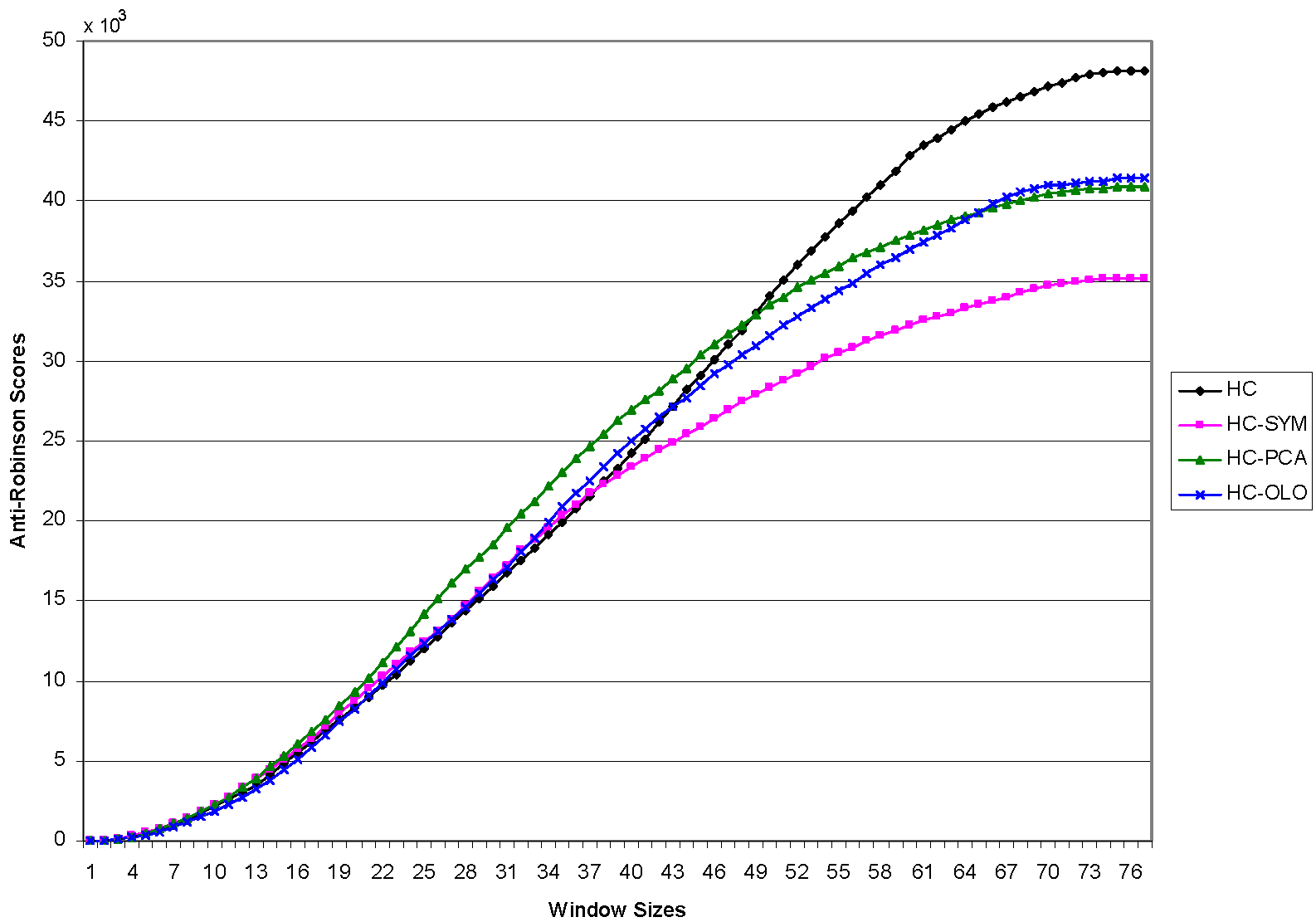

Supplement: Figure S1 — Comparison of Anti-Robinson scores for dataset A. Anti-Robnison scores by four seriation methods were compared for dataset A. (TIF) [file pone.0022546.s001.tif]

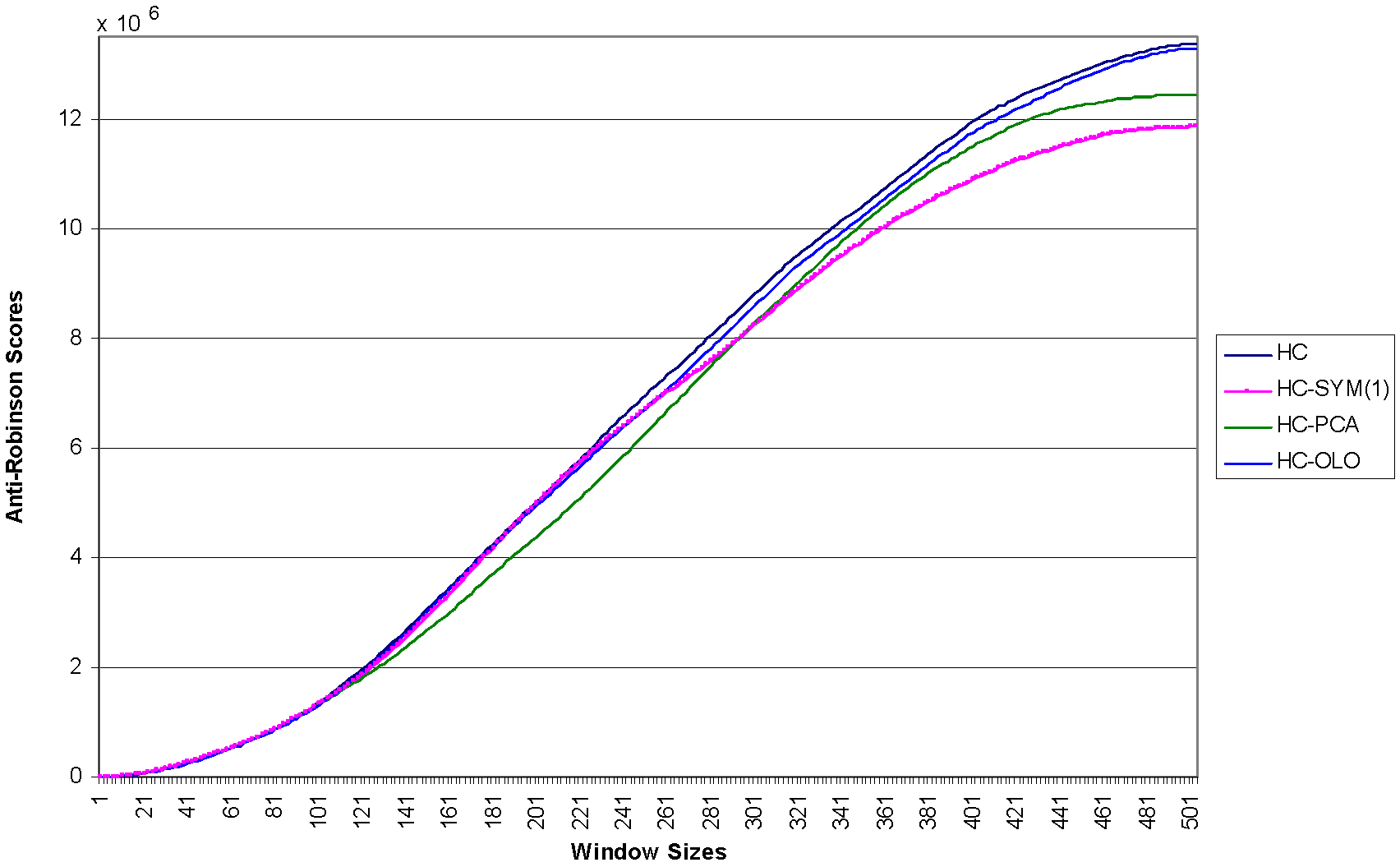

Supplement: Figure S2 — Comparison of Anti-Robinson scores for dataset B. Anti-Robnison scores by four seriation methods were compared for dataset B. The level of HC-SYM was 1. (TIF) [file pone.0022546.s002.tif]

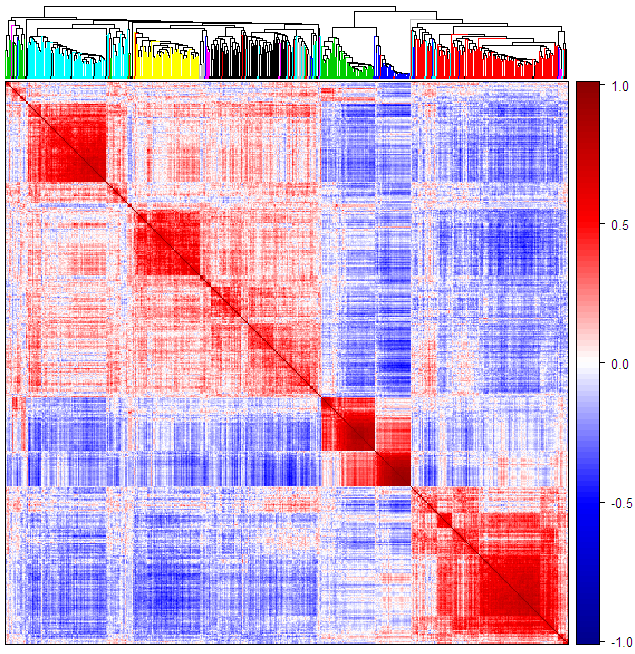

Supplement: Figure S3 — Dendrogram with proximity view for dataset B using HC. The result of HC by both dendrogram and color image of similarity matrix. (TIF) [file pone.0022546.s003.tif]

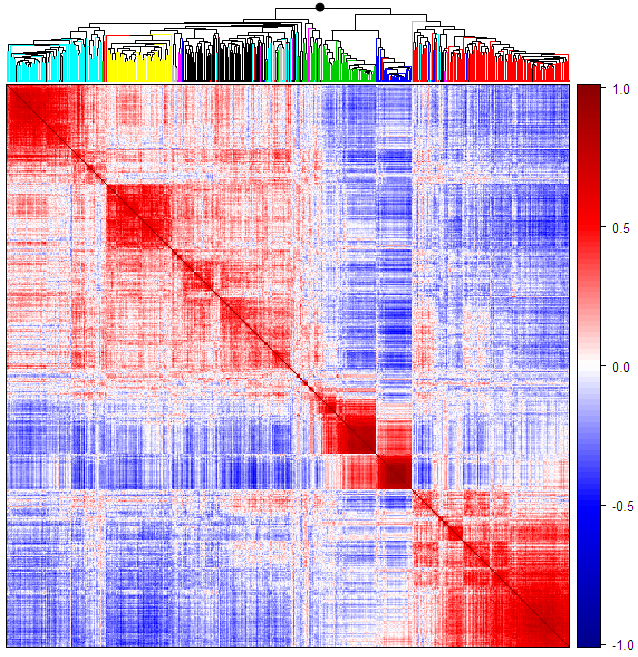

Supplement: Figure S4 — Dendrogram with proximity view for dataset B using HC-SYM. The result of HC-SYM by both dendrogram and color image of similarity matrix. The level of HC-SYM was 1. (TIF) [file pone.0022546.s004.tif]

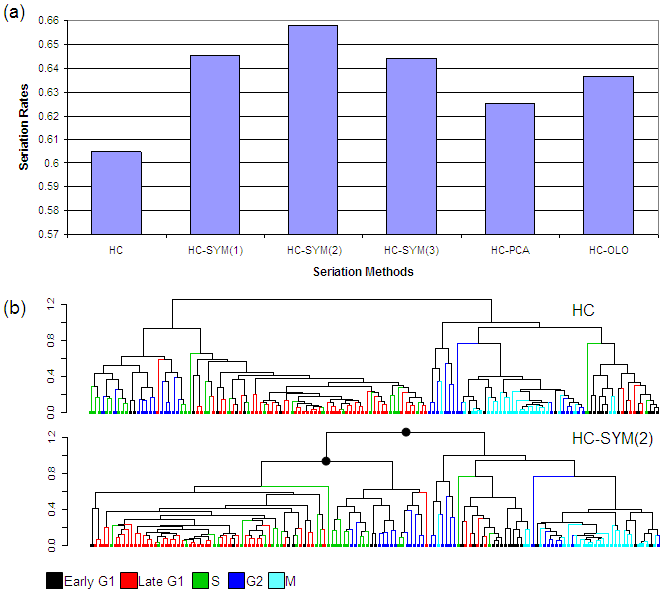

Supplement: Figure S5 — Seriation of yeast cell cycle data. The yeast cell-cycle data of Cho et al. [16] containing time-course expression profiles more than 6000 genes at 17 time points was analyzed with the proposed method using 145 genes whose phases have been assigned with a removal of one abnormal time point as suggested by Tamayo et. al [4]. (a) Comparison of seriation rates (b) Dendrogram of HC and HC-SYM at level 2. The parameter values were br = 0.3 and sr = 0.03 for HC-SYM after HC was carried by average-linkage with Pearson correlation distance. (TIF) [file pone.0022546.s005.tif]
